# Adsorptive Pattern Using Drinking Water Treatment Residual for Organic Effluent Abatement from Aqueous Solutions

**DOI:** 10.3390/ma16010247

**Published:** 2022-12-27

**Authors:** Manasik M. Nour, Maha A. Tony, Hossam A. Nabwey

**Affiliations:** 1 Department of Mathematics, College of Science and Humanities in Al-Kharj, Prince Sattam Bin Abdulaziz University, Al-Kharj 11942, Saudi Arabia; 2 Basic Engineering Science Department, Faculty of Engineering, Menoufia University, Shebin El-Kom 32511, Egypt; 3 Advanced Materials/Solar Energy and Environmental Sustainability (AMSEES) Laboratory, Faculty of Engineering, Menoufia University, Shebin El-Kom 32511, Egypt

**Keywords:** aluminum-based residue, sorption, phenol, adsorption isotherm, statistical optimization

## Abstract

Zeolite (ZSM-12) is a unique material obtained from the drinking water treatment plants’ residual “alum sludge”, as a result of using aluminum sulphate as a primary coagulant in the plants. Herein, alum sludge (AS) is initially dewatered and subjected for various calcination temperatures 400 °C, 600 °C and 800 °C and the corresponding materials are named as AS400, AS600 and AS800, respectively. Such calcination is provided to attain ZSM-12, which is considered a highly adsorptive material. The material characterization and morphology were investigated using scanning X-ray diffraction (XRD) and electron microscope (SEM) that confirm the presence of ZSM-12 and porosity of such prepared materials. Thereafter, such materials are introduced for phenol remediation from aqueous solution. The experimental data reveal that AS400 had the largest adsorption capacity (275 mg-_phenol_/g), in comparison to the commercial adsorbent materials during 2 h of isotherm time. Such a result confirms the suitability of alum sludge residue to be a good candidate for environmental remediation. Furthermore, adsorption isotherm models were applied, and the data are well-fitted to the Langmuir isotherm model. In addition, thermodynamic parameters are investigated which verify the physisorption adsorption process and exothermic nature with a spontaneous reaction system.

## 1. Introduction

As a result of demographic growth, various applications of water into agricultural and industrial sectors have become a problem, with the limited availability of fresh water. However, water could be recyclable and reusable [1,2,3]. Hence, the eventual reuse of reclaimed water appears to be a unique solution. In the majority of countries, during the last decades, wastewater treatment has become the adopted key to water crises [4]. Conventionally, hazardous organic substances categorize industrial discharge. Such ingredients include phenolic compounds [3] and aromatic intermediates [5], halogenated or volatile organic substances [6], benzene and chloroform as well as heavy metals. The existence of such materials in the discharged water causes severe toxicity and damage to water, triggering destruction to the ecosystem and thus creating problems for organisms and human beings.

Phenolic compounds are harmful to human health, causing severe problems and several health damages. Phenolic substances are also toxic to other biological species even in very low concentrations less than 1 ppm and could destroy the aquatic environment and biological species [7]. Although phenol is classified as one of the priority pollutants, phenolic compounds are considered as one of the most common industrial pollutants due to their industrial relevance. Such phenolic materials are widely employed in many industries such as petroleum refineries and petrochemical industries, coke gasifiers, wood preservation, plastics, metals, pulp and paper manufacturing and organic chemical plants as well as in agricultural activities as pesticides [8]. However, numerous research articles suggest that some of these phenolic organic compounds are recalcitrant and persist in the treated water, as they are refractory to conventional treatment [4,7,9]. Thus, for safe disposal, it is essential for these compounds to be removed from waste streams. Various techniques have been applied to minimize this serious waste. Among them, the adsorption technique is signified as an appropriate candidate since it isa cost-efficient technique [7,10]. However, the cost of commercial adsorbents is still diminishing its real applications. Generally, the most widely applied adsorbent is activated carbon. However, handling cost and the necessity for regeneration still stand as the main disadvantages of its application [9,11,12,13]. Thus, developing a green ecofriendly adsorbent approach is gaining the attention of academia and scientists to treat our waste using an industrial symbiosis approach.

On the other hand, global demand for the production of safe drinking water is associated with the generation of waterworks residuals. Alum-based waste is considered an inescapable by-product from the potable water purification plant as a result of using aluminum sulfate as a coagulant for water treatment [14,15,16,17]. However, the harmful results of aluminium exposure could be behind a variety of toxic effects in the biological environment. With the increase in environmental awareness, this worldwide waste requires appropriate management. Recently, considerable attention has been focused on preliminary studies to use alum sludge as a potential adsorbent [10,18], due to the chemical proposition and amorphous structure of the material. Such a waste stream possesses a porous nature and has a high surface area. Thus, such characteristics demonstrate it to be a significant adsorbent substance.

Although both phenolic compounds and alum sludge residuals are causing an issue to the environment, the application of aluminum-based material to be phenol adsorbent material is a beneficial treatment–environmental ecology approach. Of note, porous materials are of suggestive interest to be applied in adsorption techniques [19,20]. However, according to the best of the authors’ knowledge, there is a lack of articles cited regarding using alum sludge waste in its modified form for phenolic compound adsorption. The crucial goal of scientists is exploring environmentally benign treatment methodologies that could be acquired through an industrial ecology approach [21,22]. From this regard, aluminum-based material could be a sustainable option to produce the highly efficient adsorbent “zeolite” material. Such a technique introduces a sustainable and natural treatment solution for industrial discharge remediation. Zeolite possesses certain unique physical and chemical characteristics, making it a superior adsorbent material in water treatment application by utilizing a waste resource. Zeolite recorded a superior adsorption capacity for various pollutants such as dyes and heavy metals. Therefore, such study is leading to a sustainable engineering technology in applying an economical green sustainable technique for both wastewater remediation and waste valorization.

Herein, the present investigation explores the feasibility of various AS adsorbent materials derived from aluminum-based waterworks residue, “alum sludge”, and thermally-treated sludge at different preparation temperatures, i.e., 400 °C, 600 °C and 800 °C for phenolic compound adsorption and the adsorption process pattern was discussed. X-ray diffraction (XRD) and scanning electron microscope (SEM) and images of the prepared samples were explored. Further, adsorption thermodynamics and isotherm studies were examined.

### 1.1. Bibliometric Technique

**Figure 1:**
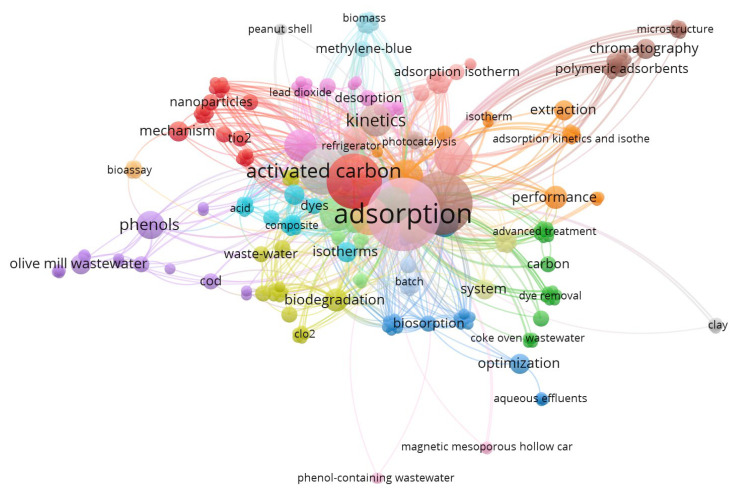
Bibliometric technique analysis for the search items “wastewater” comprising “Phenol” AND Treatment AND “Adsorption” conducted via “VOSviewer software”.

The bibliometric technique for analysis is suggested as a significant method and is applied commonly for a selected topic analysis on appropriate research literature. The bibliometric analysis tool can be used for linking key features of a specific topic. Hence, the attained bibliometric mapping categorizes the most research papers cited in a particular field in the literature. Moreover, it investigates the associated link concerning the terms accomplished. Thus, an investigation conducted using the database of the “Web of Science” utilising the keywords of “Phenol wastewater AND Treatment AND Adsorption” was performed and 1122 published articles found, in scientific research journals from the year 2000 to May of 2022. Figure 1 displays a profile of the current research work associated to the aqueous effluent comprising organic phenol adsorption over various and numerous adsorbent materials. The upsurge in the number of published articles might help in further improvement of wastewater treatment via the low-cost adsorption technique, which helps achieve a win–win sustainable environment. Further, the keywords occurrence was analyzed via “VOSviewer software” that is used to analyze the articles in such research work (version 1.6.16.0, accessed on 9 May 2022). The bibliometric network mapping produced in VOSviewer, based on 1122 articles from the examination terms, was explored in cluster plots of Figure 1. The clusters signify hotspots. In the displayed map, extensive notice can be paid to the adsorption using activated carbon, which was intensively identified among the other adsorbent materials introduced. Not only is adsorption a sustainable technique for its accessibility and no energy needs, but also the application of low-cost waste materials as adsorbents, which are recommended for a sustainable environment. Despite the availability of waste materials in every site in the whole world, up until now, the practical real treatments for adsorption using waste materials for industrial applications currently remain limited.

**Figure 2:**
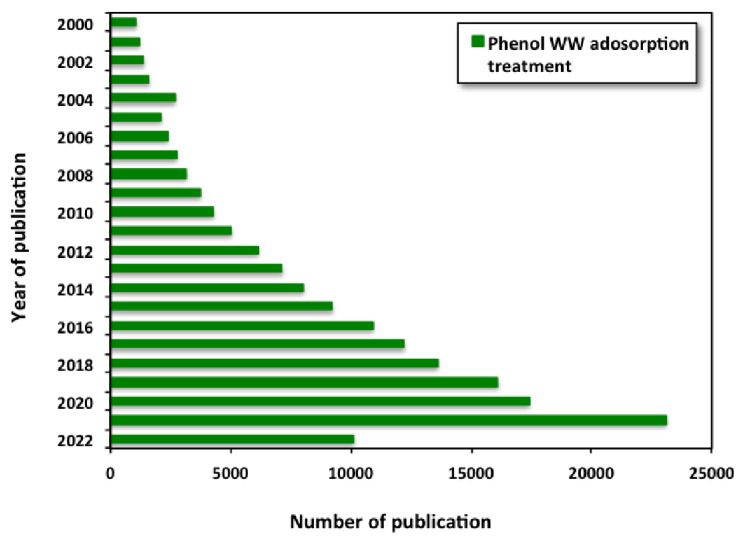
Publications on phenol wastewater treatment though adsorption technique (Data from Google scholar using the search “phenol wastewater AND treatment AND adsorption” as search terms).

Further extra data analysis was carried out to explore the growth of research focusing on phenol wastewater treatment/adsorption systems. It is noticed that there is increase in the research published through the last two decades (from 2000 to 2022), as stated in Figure 2. Hence, such studies offer the opportunity for the use of various adsorbent materials for wastewater embedded with phenol as an opportunity for further use of reclaimed water.

### 1.2. Box–Behnken Design (BBD) Model

The introduced design “Box–Behnken design” (BBD) was applied in order to explore the optimization conditions of the operating parameters of the adsorption, i.e., sample stirring (rpm) (*ξ_1_*), pH (*ξ_2_*) and AS mass (*ξ_3_*) to investigate their effects on organic material (phenol) removal. Every parameter was evaluated at three coded variables levels labeled as (−1, 0, 1), as displayed in Table 1, and the full factorial design of 15 runs was conducted with the total experimental design tabulated in Table 2. The three independent parameters, *ξ_1_, ξ_2_* and *ξ_3_*, and their mathematical correlation with the response (*Y*) could be specified by the second order polynomial equation (Equation (1)) [14,23]

(1) $$Y=\beta_{o}+\sum \beta_{i}\xi_{i}+\sum \beta_{ii}\xi_{i}^{2}+\sum \beta_{ij}\xi_{i}\xi_{j}$$

where (*Y*) is the predicted response variable and (*β_o_, β_i_, β_2_* and *β_ii_*) are the set of the independent factors that influencing the response (Y) for intercept, linear, quadratic and interaction relationships, respectively. Further, the coded optimum values of the parameters are localized via Mathematica software (version V 5.2). The statistical analysis of the data through analysis of variance, ANOVA was applied to investigate the statistical test and to evaluate the estimated model fitness [23,24]. In addition, for further analyzing the data, the statistical MATLAB R2017a software was applied to plot the response surface and contour plots.

### 1.3. Isotherm Mathematical Models

To investigate the sorption capacity of AS adsorbent substances, three isotherm models were used namely Langmuir, Freundlich and Dubinin–Radushkevich (D-R).

**Langmuir Isotherm Model:** This model assumption is based on the basis of maximum adsorption capacity of a monolayer adsorption on a homogeneous surface with the following linear form:

(2) $$\frac{C_{e}}{q_{e}}=\frac{1}{K_{L}}+\frac{a_{L}}{K_{L}}C_{e}$$

(3) $$Q_{o}=\frac{K_{L}}{a_{L}}$$

where *C_e_* is phenol concentration in aqueous solution at equilibrium (mg L^−1^), *q_e_* is the equilibrium adsorption capacity, and *a_L_* and *K_L_* are Langmuir constants n. *Q_o_* is the monolayer adsorption capacity of phenol mass adsorbed per unit mass of AS material (mg g^−1^).

**Freundlich Isotherm Model:** This model (Equation (4)) is applied for highly heterogeneous surface schemes with constant heterogeneity factor (*1/n)*.

(4) $$ln\left(q_{e}\right)=lnK_{F}+\frac{1}{n}lnC_{e}$$

where *K_F_* is a constant associated with the adsorption capacity of the solid (L g^−1^). The *1/n* is the heterogeneity constant that could express the favorability of adsorption when n value is greater than [25].

**Dubinin−Radushkevich (D-R) Isotherm Model:** This model (Equations (5) and (6)) is limited to a monolayer and applied to investigate the adsorption nature and could assess the adsorption energy [25,26,27]. Thus, it does not simulate a homogeneous adsorption surface or assuming constant sorption potential.

(5) $$lnq_{e}=lnq_{m}-K_{DR}\varepsilon^{2}$$

(6) $$\varepsilon^{2}=RTln\left(1+\frac{1}{C_{e}}\right)$$

where *q_m_* is the monolayer saturation capacity (L g^−1^) and *K_DR_* is the constant of adsorption energy that is used to estimate the average free energy (*E*) (Equation (7)), such E value verifies the chemical or physical type of adsorption type.

(7) $$E=\frac{1}{\sqrt{2K_{DR}}}$$

## 2. Materials and Methods

### 2.1. Field Sampling of Aluminium-Based Waste

On-site aluminium-based waste called alum sludge (AS) was attained from the underflow channel of the sedimentation tank in the Drinking Water Management Plant, Shebin El-Kom city in Menoufia Governorate, Egypt. The water treatment plant uses aluminium sulphate in the primary treatment process as a coagulant material and the resultant sludge in the plant is aluminium-rich sludge, as an inescapable by-product waste. Samples were taken to laboratory and kept in plastic containers prior to use and analysis. Initially, the collected sludge was subjected to gravity settling in order to remove the excess water and to concentrate the sludge before it was exposed to air-drying to produce the sludge cake. Afterwards, the sludge cake was washed then exposed to overnight drying (105 °C) in an electric furnace. The resultant dried cake was ball milled for one hour to attain a fine powder, which was called AS. Further thermal treatment was carried out for activation at 400 °C, 600 °C or 800 °C (2 h) and the corresponding AS adsorbents formed were named AS400, AS600 and AS800, respectively.

### 2.2. Additional Adsorbents

Porous activated charcoal (Norit) and silica gel (SG) were commercially obtained for comparison basis with the laboratory-prepared AS adsorbent materials. They were used as purchased with no treatment or purification.

### 2.3. Phenolic Wastewater

Phenol crystals (99% purity) were deviled by Alpha Chemicals and introduced to prepare the synthetic wastewater as a model pollutant. Primarily, 1000 ppm stock solution of phenolic solution was prepared at room temperature and afterwards a successive dilution was prepared to attain the desired phenol loadings.

### 2.4. Experimental Methodology

**Figure 3:**
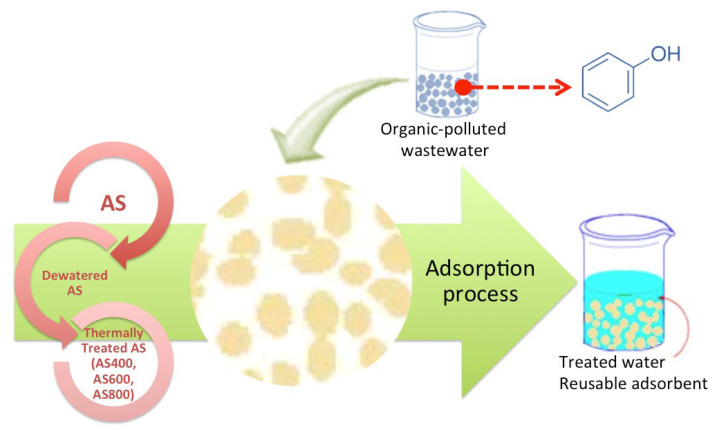
Graphical representation of the experimental set-up.

To investigate the sorption capacity of the AS-sorbents, batch experiments were conducted. AS adsorbent materials (2 g) were well-mixed with the 10-mL phenolic solution ranging from 100–1000 mg L^−1^ at a 298 K to investigate the adsorption isotherm and the samples were taken through 24 h. Thereafter, AS adsorbent materials were separated from samples before they were subjected to spectrophotometric analysis at 273 nm to estimate the remaining phenolic compound in the solution. The pH was adjusted, if required, using pre-prepared solutions used for pH adjustment that were sulphuric acid (2 M) and sodium hydroxide (10 M), which were purchased from Sigma-Aldrich. The graphical illustration of the treatment steps is displayed in Figure 3.

### 2.5. Characterization Study

The crystal structure of the various AS material (AS, AS400, AS600 and AS800) adsorbent materials was investigated by single-crystal X-ray diffraction, XRD investigation was accomplished on a Bruker-Nonius Kappa CCD diffractometer with CuKα radiation source (λ = 1.5406 Å). This measurement was carried out using a diffractometer that worked at 45 kV with a step scan time of 18.87 s. The XRD explored under step–scan mode and the registered intensities of the diffracted X-rays were detected every 0.026° over 2θ range of 20–45°. Moreover, the morphologies of the arranged sludge samples were investigated and imaged by field-emission scanning electron microscope with typical magnifications of ×8000 and ×60,000. (SEM) (FE-SEM, Quanta FEG 250, FEI Company, Hillsboro, OR, USA).

## 3. Results and Discussion

### 3.1. Characterization of AS adsorbent Materials

**Figure 4:**
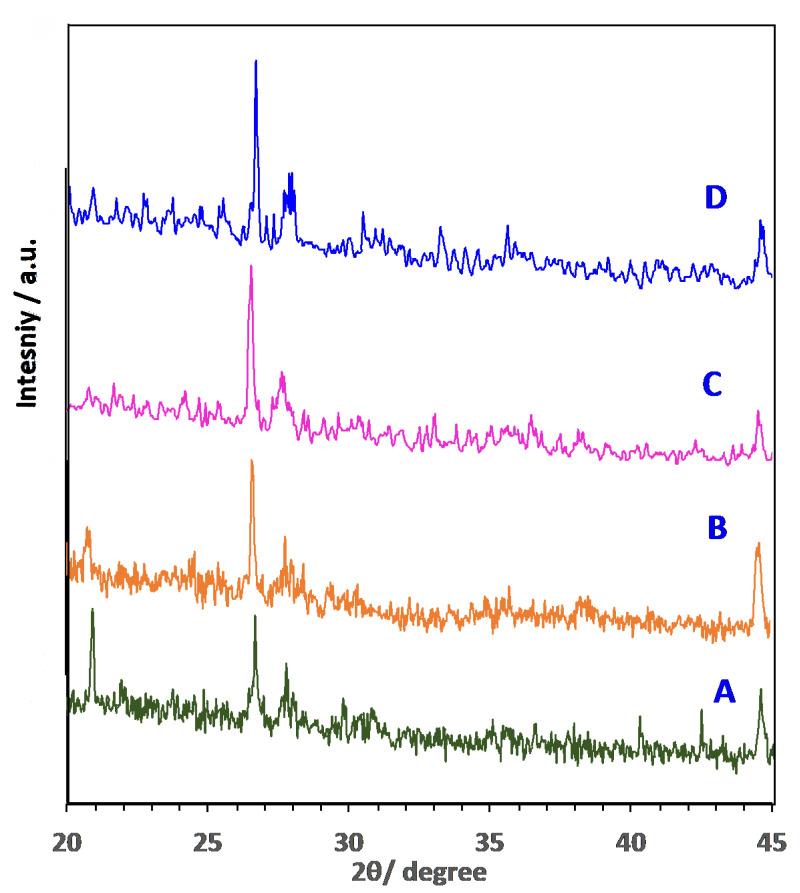
XRD diffraction pattern of AS adsorbent substances: (**A**) AS; (**B**) AS400; (**C**) AS600 and (**D**) AS800.

X-ray diffraction of the phase formation for the various AS adsorbent materials was examined using a diffractometer. The XRD in Figure 4 indicates the crystalline type of the samples. The results showed that for the alum sludge and the modified treated sludge (AS400, AS600 and AS800), the dominant formed crystalline inorganic matter is hexagonal quartz, SiO_2_ since aluminium sulphate is used in such drinking waterworks facility as a primary coagulant material. The suspended materials, such as sand and clay in the influent water used as a basis of treated water, could be the source of such quartz material. The most intense peaks of SiO_2_ are allocated at the preferred planes [100], [101], [110]. Moreover, anorthic crystal structure of calcium aluminoscilicate (CaAl_2_Si_2_O_8_) emerges in the all-prepared samples in the XRD graphs; however, its significant amount increases in the calcinated sludge and allocated with its planes [-110], [0–21] and [0–22s]. Further, ZSM-12, rich-silica zeolite (Na1.16Al2Si77.4O158.38) material crystal structure emerges especially in the calcined sludge (400 °C). Generally, SiO_2_ and Al_2_O_3_ are signified the chief zeolite precursors that are rich in the alum sludge, due to the existence of sand and using Al_2_(SO_4_)_3_ in the waterworks plant with the presence of NaOH. Such precursors presence, in addition to the calcination to 400 °C, results in the formation of zeolite (ZSM-12), which is signified by its peaks [110], [002] and [010]. This zeolite formation is affected by the ratio of the SiO_2_ and Al_2_O_3_; also, the loss of ignition [28]. Moreover, a remarkable upsurge in the quartz amount is attained by increasing the calcination temperature, which is associated to the crystallization temperature of amorphous silica [4,29]. Therefore, it is logical to assume high adsorption uptake could be attained in the sample that possesses the zeolite (ZSM-12), which is linked to the AS400 sample. This assumption belongs to the high adsorption capacity of the zeolite, which is signified as a preferable adsorbent material. It is noteworthy to mention that the presence of zeolite helps in increasing the adsorption uptake. The lattice structure of such material provides an extensive interior and exterior surface area that signifies this material possesses the tendency of ion exchange and chemical reactions. These pores could act and service as a molecular sieve. Zeolites are naturally anions and they have capacity for high ion exchange [30].

**Figure 5:**
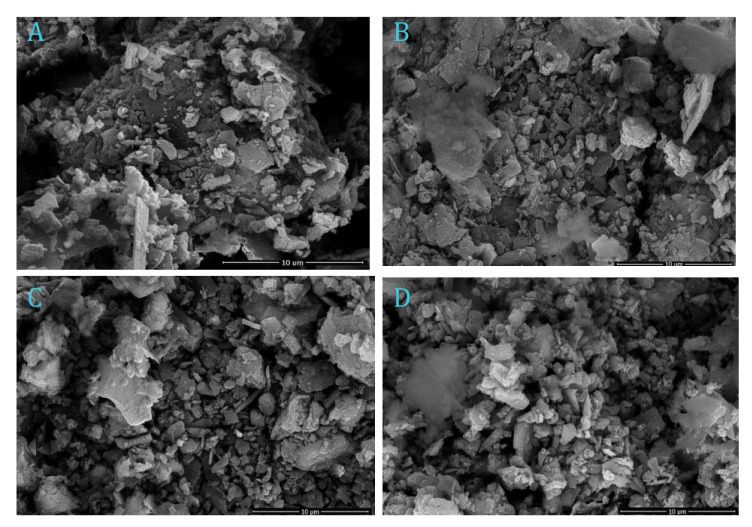
SEM micrograph of alum sludge (**A**) AS and calcined alum sludge (**B**) AS400, (**C**) AS600 and (**D**) AS800.

In order to study the material’s surface, the morphologies images aspect by field-emission scanning electron microscope, SEM, of the AS materials (AS, AS400, AS600 and AS800) are displayed in Figure 5. The surface structure of the prepared AS materials signifies the surface porosity, which is heterogeneous in nature and possesses a porous structure and non-uniform surface mixture (Figure 5A). Hence, alum sludge has an uneven structural nature. In addition, the calcined sludge morphologies images at various temperatures, namely AS400, AS600 and AS800 are represented in Figure 5B–D), respectively. It is notable that, in all samples, the porous structure with various size of porous sheets is attained. This confirms the accessibility of adsorbing phenol molecules from wastewater solution. It is obvious that calcination of sludge is associated with chemical reactions involving the dehydration of colloidal Al(OH)_3_, in addition to the appearance of Al_2_O_3_, and crystallization process to the amorphous silica. Such reactions lead to an effect on the sludge microstructure. Calcination of sludge at 400 °C represents the amorphous nature of silica (Figure 5B); however, the higher temperature of the treatment leads to the crystallization and combination of amorphous silica [31], as displayed in Figure 5C,D.

Overall, it could be concluded from such micrographs that it is possible to observe the porous structure is an evident way for phenol adsorbate material to access the adsorbent material and retain it from the aqueous media, presumably leading to formation of a monolayer or molecular cloud of the phenol molecules over the adsorbent AS surface.

In addition, the crystallite size of the prepared material was investigated and the size is in micro-particles size within the range of 0.181 to 1.8 μm and recorded a predominant particle size of 0.545 μm.

### 3.2. Assessment of Organic Effluent Removal by AS Adsorbent Materials

#### 3.2.1. Contact Time for Phenol Adsorption

**Figure 6:**
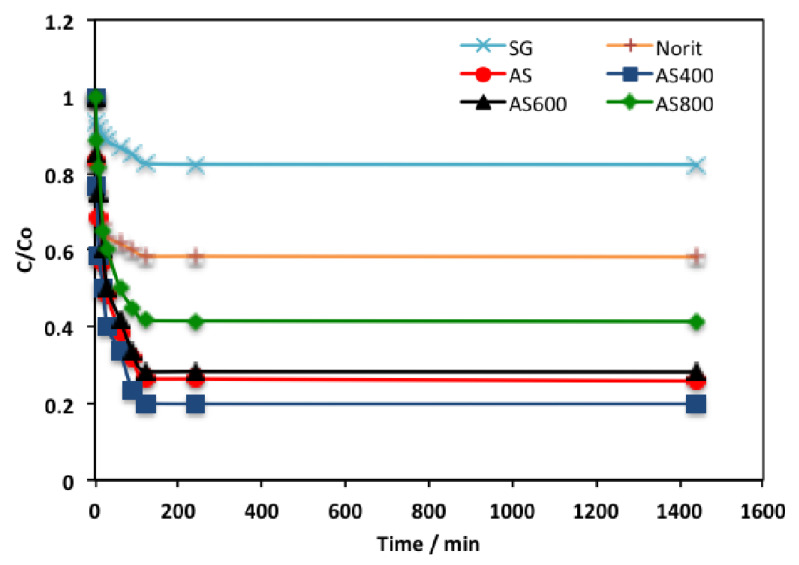
Contact time profile on the phenol uptake using different adsorbents.

Initially, to design the adsorption system, it is essential to explore the time of adsorption equilibrium. The time outline of organic effluent adsorption using AS adsorbents (AS, AS400, AS600, AS800), in addition to the two common commercial adsorbent materials (activated carbon called Norit and silica gel called Sgel) for comparison was investigated at room temperature and displayed in Figure 6. To absolutely attain the equilibrium time, up to 24 h time was applied for each adsorbent. The results revealed that, overall, AS400 displayed the highest phenol adsorption capacity, with the majority of the phenol adsorbed through the first 2 h. To add up the required time to achieve equilibrium for all the adsorbents was 2 h. Both Norit and SG showed the lowest phenol uptake. However, AS adsorbents, AS400, showed the maximum phenol removal. An explanation may be associated with the surface area and pore size distribution [32]. Thus, the presence of the ZSM-12 in the thermally treated sludge shows its significance in enhancing the adsorption capacity. Zeolite (ZSM-12) seems to be a cation exchanger because of the negative charge of its outline. Hence, ZSM-12 attached and bounded to the water molecules and then the phenol molecules are adsorbed. Other related studies also confirmed the importance of zeolite in wastewater treatment [33,34,35].

#### 3.2.2. Multivariate-Parameters Effect on Adsorption Capacity

**Figure 7:**
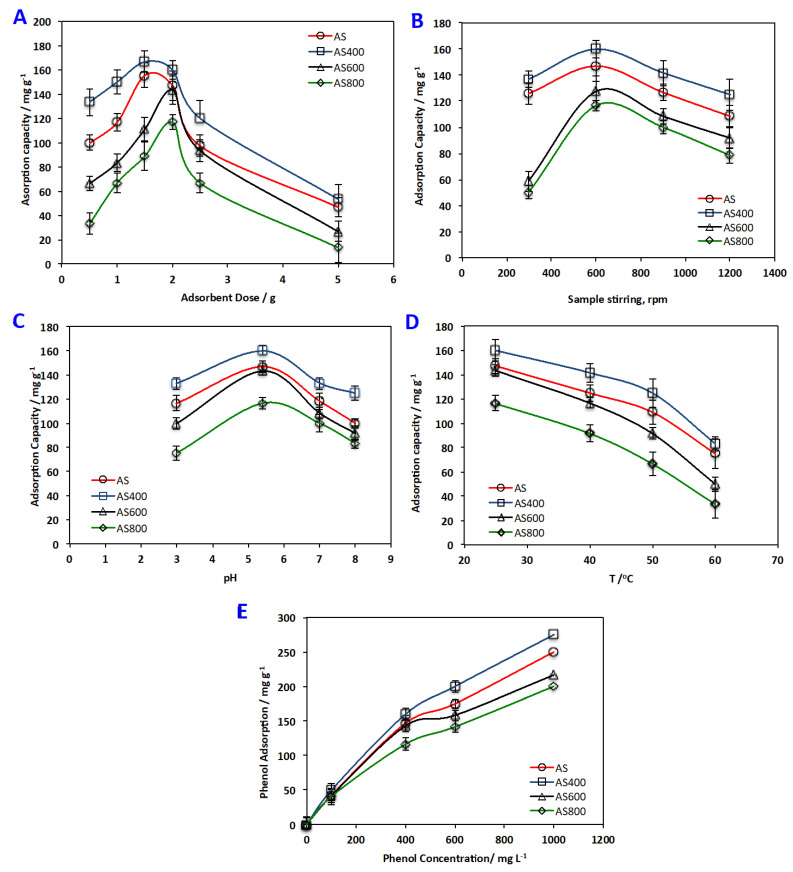
Effect of (**A**) adsorbent dose(**B**)sample stirring (**C**) pH (**D**) temperature and (**E**) phenol concentration.

Figure 7A demonstrates the effect of AS material dosing on the phenol uptake. The adsorption capacity increased with elevating the sorbent dose up to 2 g L^−1^. This could be associated with the assumption that increasing the sorbent material increases the available sportive active sites, but further increase of the sorbent material may cause particulate interaction such as aggregation that declines the total available surface area for adsorption. Further, overlap of adsorption sites may occur that account for the small increase in percentage of phenol adsorption [36,37,38].

Scattered research articles were studied on investigating the effect of mixing speed of the aqueous effluent containing organic pollutant and the adsorbent. AS materials were added to the solution while the agitating speeds were changed from 300 to 1200 rpm, keeping all the other variables constant. The influence of mixing tendency on the degree of adsorption is exhibited in Figure 7B. At exit time, phenol elimination enhanced with the mixing speed increased. This may be attributed to higher speeds of mixing; a good contact is attained concerning the adsorbent and adsorbate. Similar previous works are in accordance with such results in treating fluoride using activated charcoal as an adsorbent [39]. However, vigorous mixing is not needed since it helps the phenol molecules to leave the adsorbent surface [38,40].

The influence of pH of aqueous solution on the adsorption capacity of alum sludge adsorbent materials for organic material removal from aqueous effluent is displayed in Figure 7C. As it is shown in the Figure 7C that the extent of phenol adsorption on the materials was increased with increasing pH, nevertheless, there was a further pH increase, lessening sorption capacity. The maximum adsorption was reported at a pH of 4.5 and the values are 147, 160, 143 and 117, that correspond to AS, AS400, AS600 and AS800, respectively. This may be attributed to the pH effect on the surface charge of the adsorbent materials. At low pH values, AS materials’ surface is positively charged. This leads to the formation of static repulsion forces. However, phenol adsorption increases by pH increase due to the decrease of static repulsion forces. Although further pH increase resulted in a decrease in phenol uptake, this may be because at higher pH (i) repulsion forces formed between the phenol ions adsorbed themselves, and (ii) increase in negative charge of AS adsorbent surface and phenol converted from molecular state to ionic state that creates a repulsion force between phenol ions and AS adsorbent materials [41,42]. Therefore, the pH of the phenolic solution determines the surface charge of the adsorbent and the state of adsorbate in solution. Thus, the original solution of phenol pH (5.4) was designated to be the optimum pH.

The temperature effect on the AS adsorbent/phenol sorption system was studied at four different temperatures, i.e., 25 °C, 40 °C, 50 °C and 60 °C (Figure 4D). For all the AS adsorbent materials used, phenol uptake decreased by increasing temperature, indicating the AS adsorbent materials/phenol sorption system was apparently exothermic. AS400, which had the highest adsorption trend, showed a decrease in the adsorption capacity from 160 to 83 mg g^−1^ by temperature increase from 25 to 60°C, respectively. Such results could be associated with the temperature increase, leading to weakening of the attractive forces of adsorption between the AS materials surface and the phenol molecules. The result is a decline in the overall adsorption process. Such an investigation was recorded previously by scattered researchers [43,44]

The adsorption capacity of different phenol concentrations, ranged from 100 to 1000 mg L^−1^, onto AS-sorbents materials is illustrated in Figure 4E. The results revealed that AS400 showed higher phenol adsorption capacity overall; adsorption capacity enhanced from 50 to 275 mg g^−1^ with the phenol upsurge from 100 to 1000 mg L^−1^, respectively, as a result of enhancement in interaction between the phenol species and AS adsorbent that presented at higher concentrations. This could be related to the mass transfer zone increasing with the increase in phenol concentrations and thus the adsorption uptake is enhanced due to the high loading rate [45,46].

#### 3.2.3. BBD Factorial Design and ANOVA Testing:

The three most independent analytical variables effects in the adsorption system, sample stirring, pH and AS mass, on phenol removal were simulated by applying a Box–Behnken design model, BBD, that is displayed in Table 2. The data assigned from the model design were accessed and fitted to a quadratic polynomial model equation. Then, the model was validated through the quadratic polynomial equation via the response of phenol removal (%Y) according to the following relation:

(8) $$Y=55.19+1.23\zeta_{1}+1.66\zeta_{2}+11.54\zeta_{3}-10.33\zeta_{1}^{2}-0.3\zeta_{1}\zeta_{2}+0.53\zeta_{1}\zeta_{3}-2.53\zeta_{2}^{2}+0.21\zeta_{2}\zeta_{3}-8.02\zeta_{3}^{2}$$

SAS software was applied and the models’ multiple parameters statistical analysis (ANOVA) permit calculation of the connection between the examined parameters and the maximal phenol elimination response. Table 3 demonstrates the ANOVA data. The results tabulated in Table 3 exposed that the quadratic polynomial model is very suggestive with an accepted correlation coefficient value, *R*^2^. Generally, according to the statistics determination, the model is acceptable if the coefficient of determination, *R*^2^ is greater than 80%. Additionally, the quadratic model is well-validated when a reasonably large value of the Fisher test is explored that is superior to unity; also, a smallest *p-*value (<0.05) is attained. The determination coefficient value of the suggestive model is 98.7%, that indicates the fitness of the achieved model. As low *Pr > F* value is achieved at 0.0003, the ANOVA data explore a great correlation between the response and the variables. Therefore, it could be proposed that, from the ANOVA test, the suggestive model well correlates the results.

**Figure 8:**
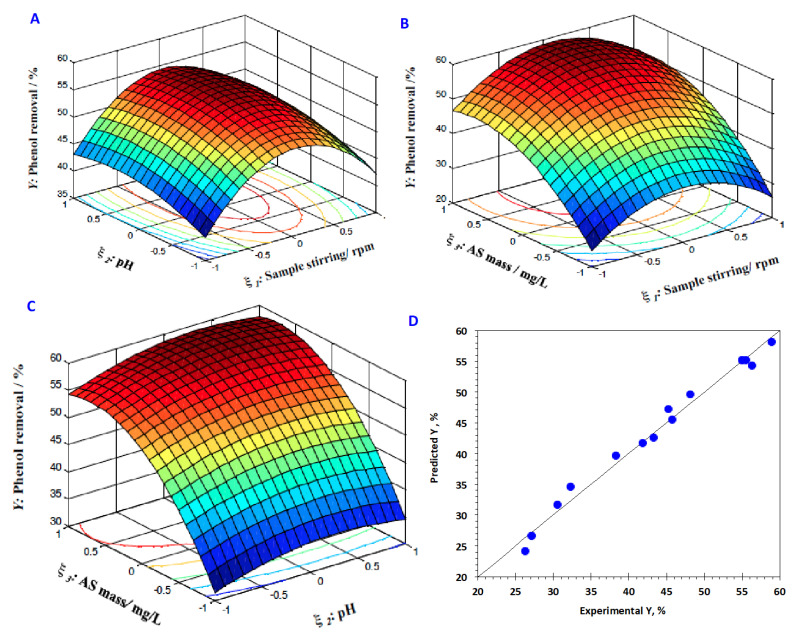
3D Surface conjugated with 2D contour graphs of the RSM of phenol adsorption, (**A**) sample stirring vs. pH; (**B**) sample dose vs. pH, (**C**) sample stirring vs. sample dose and (**D**) graphical plots of the experimental and predicated responses.

To further illustrate the results, the interactions between the examined variables, sample stirring, pH and AS dose, are necessary to be recognizable. Hence, the 3D surface and 2D contour plots are drawn to explain the examined regression model and the plots are exhibited in Figure 8A–C). The graphs illustrate the interaction effect of each two independent variables investigated on the phenol elimination.

As investigated in Figure 8A, phenol adsorption efficacy that is confirmed via its adsorption removal percent is steadily enhanced with the elevation in both the reagent dosage of both reagents stirring of sample and pH of the aqueous effluent. The chief reason of such a trend is related to the phenol removal, which is increased with increase of the sample stirring and the pH of the medium. However, behind a specific catalyst limit, phenol elimination is deduced. Such a suggested relation might be associated with the repulsion force between phenol ions and AS adsorbents at low pH value. The sample stirring is important to attain the highest contact between the phenol molecules and the AS materials. Furthermore, the curvature of the surface plot of the 3D graph is an extent for the degree of extravagant on the response (*Y*%), since the higher circular contour curvature signifies a weaker interaction outcome. In addition, as seen from Figure 8B,C, it is noted that the reaction is very sensitive to the pH variation, in comparison to the other variables investigated. To add up, Equation 8, the quadratic model equation, is affected to explore the relation between the predicted and experimental responses that illustrate the values attained that were close to linearity, which confirms a measurable way for the data that was accurate and dependable consistent with the results presented in Figure 8D.

Additionally, Mathematica software (version V 5.2) was applied to investigate the numerical simulated optimization of the system further attained and the optimum values recorded are 614 rpm, 5.3 pH and 1.9 g of AS adsorbent, respectively, at 60% removal. Additionally, in order for results verification, the explored expected optimal values were applied to conduct further experiments through duplicates of experiments and original response values are compared with the simulated ones, that reached phenol removal of 62%, with a high relationship between the predicted and experimental model that confirms the best fit of the model.

#### 3.2.4. Adsorption Isotherm Models

To further explore the mechanism of the sorption process of phenol molecules on the AS substances, the experimental results was used as a function of isotherm models. The experimental results were applied to Langmuir [47], Freundlich [48] and Dubinin–Radushkevich (D-R) [49] isotherm models to evaluate AS-sorbents/phenol adsorption mechanism. The constant parameters of those isotherm equations were analyzed through regression of the linearized form of the models’ equations. The models’ constant parameters, together with the correlation coefficient (*r*^2^), are tabulated in Table 4.

From the correlation coefficient values, *r*^2^, all the AS-sorbents display a good correlation with both Langmuir and Freundlich models, which means the adsorption process through those materials is going beyond monolayer coverage which, once saturated with phenol, would penetrate the multilayers. The mean free energy values from the D-R model confirm the phenol adsorption process onto AS-sorbents is a physical adsorption.

#### 3.2.5. Thermodynamic Investigation

In order to well-explore the sorption mechanism of phenol onto alum sludge adsorbents system, thermodynamic variables, i.e., enthalpy, Gibbs free energy, and entropy changes were assessed as their standard equations [32] (Table 5).

**Figure 9:**
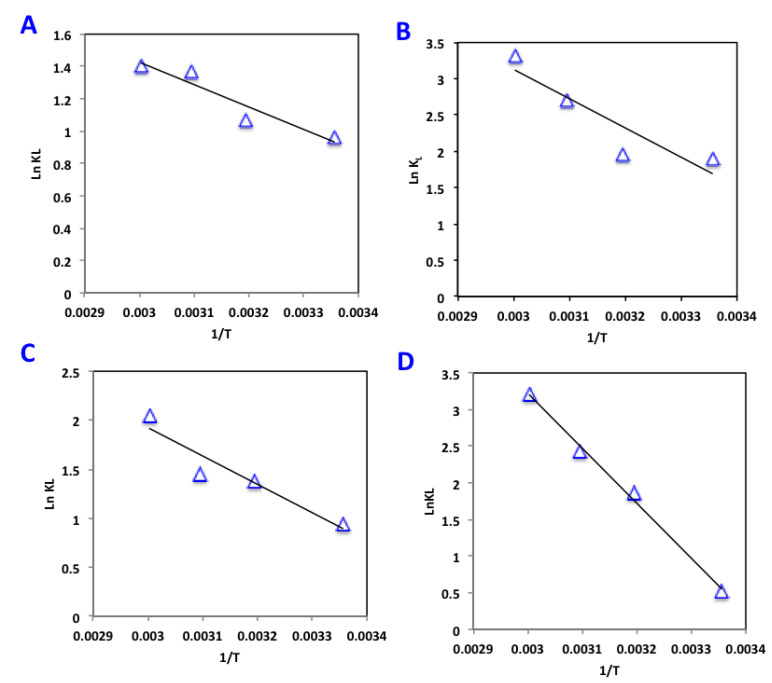
Van’t Hoff plot of lnKL vs. 1/T(K) for phenol adsorption on AS-based materials (**A**) AS, (**B**) AS400, (**C**) AS600 and (**D**) AS800.

The Van’t Hoff equation was used to investigate the values of enthalpy (ΔH) and entropy (ΔS) as given in Equation (9). Since, the equilibrium constant (*K_L_*) can be used to investigate the thermodynamic variables, the change in Gibbs free energy of the sorption is attained by Equation (10) [25].

(9) $$lnk_{L}=\frac{\Delta S}{R}-\frac{\Delta H}{RT}+$$

(10) $$\Delta G=-RTlnk_{L}$$

where *R* is universal gas constant (8.314 J/mol K) and *T* is the absolute temperature (K). Thus, the change in enthalpy (∆*H*) and entropy change (∆*S*) are attained with the plot of ln*K_L_* against 1/*T*, since their calculations are given from the slope and intercept of such plot, respectively (Figure 9).

Across all temperatures, all alum sludge adsorbent materials presented negative values for both Gibbs free energy and enthalpy. Such results indicate the spontaneous and exothermic nature of the adsorption system. However, positive entropy change values indicate an unequal upsurge in the degree of randomness at the solid–adsorbate interface during the system of phenol sorption onto alum sludge adsorbent materials.

Comparing the AS400 to the commercial Norit and SG, a superior adsorption capacity is attained due to better dispersion interaction existing on the surface of AS400 rather than commercial adsorbents, which are increased by the physical adsorption. Thus, phenol adsorption uptake performance on the given porous material is related to the chemical composition and the porous features of the adsorbents. Hence, phenol uptake is not only associated with the material porosity, but it is also associated with the terms of surface chemistry of the material. This may be associated with the stronger hydrophobicity on the surface of AS after modification. In addition, the adsorption system was sensitive to the change of the solution pH.

A comparison of the phenolic compounds removal, using adsorption methodology utilising various adsorbent materials from several studies cited in the literature and comparing them with the current investigation, is tabulated in Table 6. It can be concluded that the current study showed a superior adsorption capacity compared to the other materials. Although other adsorbents also showed a high adsorption capacity, such as coconut shell material, the current investigation still revealed a reasonably high adsorption capacity. It is also noteworthy to mention that using ZSM-12 derived from aluminum-based waste possesses double benefits since it is converting a toxic aluminium containing waste into valuable ZSM-12 zeolite type material. Thus, it destroys harmful aluminium-based waste and eliminates another pollutant material. However, in the case of other adsorbents, such as coconut shell, it is only a waste, neither toxic nor harmful to the environment. Hence, this study introduces an industrial symbiosis investigation.

#### 3.2.6. Adsorbent Stability and Reusability

**Figure 10:**
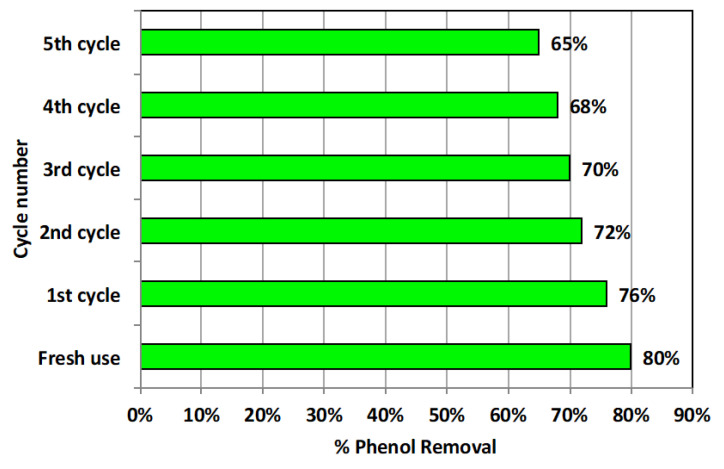
Adsorbent cyclic use activity in phenol removal.

After treatment, the adsorbent material was washed out for reuse in order to investigate its cyclic performance and treatment efficiency. In this regard, recovered alum sludge adsorbent material was collected after treatment and subjected for successive distilled water washing for regeneration facility. Then, the resultant material was oven dried (for 1 h at 105 °C) to be ready for use in successive treatment. The outcome of this work is illustrated in Figure 10. As displayed in Figure 10, the alum sludge material showed a good reusability potential since its removal efficiency reached 65% removal after the fifth cycle use, compared to 80% removal for the fresh use. Furthermore, the aluminum release in water is measured in the treated water; only 0.003 g L^−1^ of aluminum is monitored after treatment in the wastewater according to use of 2 g L^−1^ of alum sludge material. These results confirm the AS stability and its adsorption activity even after several uses. These data suggest such material to be a suitable candidate for real industrial applications. However, further research could be conducted for industrial large-scale application. In this context, appropriate material characteristics must be optimized for design aspects. Thus, upgrading such a procedure could highlight a new opportunity for a sustainable world in the modern era.

## 4. Conclusions

Organic pollutant remediation from aqueous effluent was successfully removed through adsorption technique using low-cost waste materials residue from waterworks plants. Phenol adsorption from an aqueous solution utilised various adsorbent substances involving commercially available Norit and Sicilia gel as well as four novel alum-sludge-derived materials, AS, AS400, AS600 and AS800. AS400 revealed the highest phenol adsorption capacity that reached to 275 mg-_phenol_/g during 2 h of isotherm time. The adsorption isotherm data revealed that the results were well-fitted with the Langmuir model, which possessed the highest correlation coefficient. Meanwhile, phenol uptake decreased with temperature increase. In addition, response surface methodology based on Box–Behnken design revealed the optimal operating variables that were recorded as 614 rpm, 5.3 pH and 1.9 g of AS adsorbent material. Thermodynamic variables investigated the spontaneous and exothermic adsorption system. This modified waste material displays excellent promise for a cheap, simple and effective method for phenol removal from waste streams.

## Figures and Tables

**Table 1:** Levels of coded and natural parameters range applied in RSM model.

Variable	Symbols		Range and Levels		
	Natural	Coded	−1	0	1
Sample stirring (rpm)	e*_1_*	ξ*_1_*	400	600	800
pH	e*_2_*	ξ*_2_*	4.0	5.0	6.0
AS mass (mg/L)	e*_3_*	ξ*_3_*	1.0	1.5	2.0

**Table 2:** Box–Behnken design model for factorial design of phenol adsorption experiments optimization.

Experimental Runs	Factors		
	ξ*_3_*	ξ*_2_*	ξ*_3_*
1	−1	−1	0
2	−1	1	0
3	1	−1	0
4	1	1	0
5	0	−1	−1
6	0	−1	1
7	0	1	−1
8	0	1	1
9	−1	0	−1
10	1	0	−1
11	−1	0	1
12	1	0	1
13	0	0	0
14	0	0	0
15	0	0	0

**Table 3:** ANOVA statistics test for the BBD model*.

Source	Degree of Freedom (df)	Sum of Squares (SS)	Mean Squares (MS)	Fisher *F*-Values	Probability *p*-Values
**Model**	9	1700.22	188.910	42.179	0.0003
**Linear**	3	1099.94	1099.93	245.58	0.2387
**Square**	3	660.870	660.868	147.55	0.0710
**Interaction**	3	0.65550	1.65555	0.3693	2.2761
**Error**	5	22.3943	4.47887		
**Total**	14	1722.60			

* *R*^2^ = 98.70%; adj*R*^2^ = 96.36%.

**Table 4:** Isotherm parameters for organic pollutant adsorption on different alum sludge materials *.

Adsorbent Material	Adsorption Model										
	Langmuir				Freundlich			Dubinin—Radushkevich			
	*a_L_* (L/mg) ×10^−2^	K_L_	*Q_o_* (mg/g)	*r* ^2^	*K_F_*	*n*	*r* ^2^	*q_m_* (mol/g)	*K*′ (mol^2^/J^2^) ×10^−4^	*E* (kJ/mol)	*r* ^2^
**AS**	0.89	2.62	294.12	0.98	1.99	1.92	0.97	188.67	0.60	0.091	0.92
**AS400**	2.40	6.74	285.71	0.98	3.34	42.67	0.71	5.51	0.01	0.707	0.91
**AS600**	1.04	2.56	243.9	0.94	2.18	12.85	0.79	172.36	0.70	0085	0.94
**AS800**	0.72	1.68	232.56	0.98	2.22	11.11	0.91	150.22	0.80	0.079	0.90

* *aL*, K_L_: Langmuir adsorption constants; *Qo*: monolayer adsorption capacity; *KF*: Freundlich constant; *n*: heterogeneity constant; *q_m_*: monolayer saturation capacity; *K′*: D-R isotherm constant; *E*: sorption mean free energy.

**Table 5:** Thermodynamic parameters for phenol adsorption by different alum sludge materials.

Adsorbent	ΔG (KJ mol^−1^)				ΔH (KJ mol^−1^)	ΔS (J mol^−1^ K^−1^)
	25 °C	40 °C	50 °C	60 °C		
**AS**	−2.38	−2.78	−3.67	−3.89	−11.51	46.37
**AS400**	−4.73	−5.12	−7.28	−9.17	−33.54	126.64
**AS600**	−2.33	−3.58	−3.90	−5.68	−24.02	88.06
**AS800**	−1.29	−4.84	−6.52	−8.89	−62.38	213.93

**Table 6:** Comparison of adsorption capacities of various adsorbents with the current study for phenolic compounds removal.

Adsorbent	Chromium Metal	Adsorption Capacity	Temperature, K	Ref.
Zeolite (ZSM-12) from aluminium waste	Phenol	275	298	Current study
coir pith carbon	2-chlorophenol	18	298	[53]
Lignin from black liquor	Bisphenol-A	85	298	[51]
Starbon (from natural polysaccharides)	Phenolic compound	87	298	[54]
Carbonized biological sludge	phenol	50	298	[50]
Coconut coir pith	phenol	37	303	[11]
Straw	p-chlorophenol	129	294	[52]
Coconut shell	p-chlorophenol	334	294	[52]

## Data Availability

Data are available upon request.
